# Post-operative stress hyperglycemia is a predictor of mortality in liver transplantation

**DOI:** 10.1186/s13098-018-0334-5

**Published:** 2018-04-19

**Authors:** Elena Giráldez, Evaristo Varo, Ipek Guler, Carmen Cadarso-Suarez, Santiago Tomé, Patricia Barral, Antonio Garrote, Francisco Gude

**Affiliations:** 1 Intensive Care Unit, Hospital Clínico Universitario de Santiago, Travesia da Choupana s/n, 15706 Santiago, Spain; 2 Abdominal Transplantation Unit, Hospital Clínico Universitario de Santiago, Santiago, Spain; 3 Biostatistics Unit, Department of Statistics and Operations Research, University of Santiago de Compostela, Santiago, Spain; 4 Clinical Epidemiology Unit, Hospital Clínico Universitario de Santiago, Santiago, Spain

**Keywords:** Stress glycaemia, Diabetes, Mortality, Glucose profiles, Joint modelling

## Abstract

**Background:**

A significant association is known between increased glycaemic variability and mortality in critical patients. To ascertain whether glycaemic profiles during the first week after liver transplantation might be associated with long-term mortality in these patients, by analysing whether diabetic status modified this relationship.

**Method:**

Observational long-term survival study includes 642 subjects undergoing liver transplantation from July 1994 to July 2011. Glucose profiles, units of insulin and all variables with influence on mortality are analysed using joint modelling techniques.

**Results:**

Patients registered a survival rate of 85% at 1 year and 65% at 10 years, without differences in mortality between patients with and without diabetes. In glucose profiles, however, differences were observed between patients with and without diabetes: patients with diabetes registered lower baseline glucose values, which gradually rose until reaching a peak on days 2–3 and then subsequently declined, diabetic subjects started from higher values which gradually decreased across the first week. Patients with diabetes showed an association between mortality and age, Model for End-Stage Liver Disease score (MELD) score and hepatitis C virus; among non-diabetic patients, mortality was associated with age, body mass index, malignant aetiology, red blood cell requirements and parenteral nutrition. Glucose profiles were observed to be statistically associated with mortality among patients without diabetes (*P *= 0.022) but not among patients who presented with diabetes prior to transplantation (*P *= 0.689).

**Conclusions:**

Glucose profiles during the first week after liver transplantation are different in patients with and without diabetes. While glucose profiles are associated with long-term mortality in patients without diabetes, after adjusting for potential confounding variables such as age, cause of transplantation, MELD, nutrition, immunosuppressive drugs, and units of insulin administered, this does not occur among patients with diabetes.

## Background

Orthotopic liver transplantation (OLT) is the established treatment for end-stage liver disease and acute fulminant hepatic failure, and more than 100,000 OLTs have been performed in Europe [1]. Advances in both medical management and surgical techniques have led to an increase in the number of long-term survivors [2].

Alterations in glucose metabolism are common among patients undergoing surgery, and are associated with increased risk of mortality and morbidity [3]. These abnormalities, particularly hyperglycaemia, are also common in critically ill patients, even those without a diagnosis of diabetes. Hyperglycaemia associated with critical illness is commonly regarded as an extreme form of “stress hyperglycaemia”, typically attributed to insulin resistance caused by endogenous and exogenous catecholamines and glucocorticoids. High levels of circulating free fatty acids inhibit peripheral glucose uptake and use, causing hepatic steatosis, which impairs liver glucose regulation in the critically ill [4, 5]. In addition, blood glucose concentrations vary markedly in critically ill patients, even when using continuous feeding and insulin infusions, and blood glucose values and incidence of hyperglycaemia have a circadian rhythm in critically ill patients [6]. Indeed, blood glucose variability can be quite different in the presence of the same mean blood glucose value.

Several researchers have consistently reported a significant association between increased glycaemic variability and worse outcome in critically ill patients [7–12]. In their analyses, blood glucose variability is measured by using standard deviation, percentile values and successive changes in blood glucose, and by calculating the coefficient of variation. It is, however, recognised that, compared to the use of only single-moment biomarker values, serial biomarker evaluations may carry important additional information as regards prognosis of the disease under study.

We hypothesised that postoperative blood glucose concentrations would independently predict mortality in patients undergoing OLT. During patients’ stay in hospital, clinicians use longitudinal glucose measurements to gain a better understanding of disease dynamics. Accordingly, this study sought to investigate the ability of postoperative glucose profiles measured for 7 days to predict mortality in patients who underwent OLT, differentiating between those with and those without pre-existing diabetes mellitus. The relationship between glucose profiles and risk of death was modelled using flexible joint modelling of longitudinal data and time-to-event analysis.

## Patients and methods

We conducted an observational study of 642 patients undergoing liver transplantation at a single, tertiary care transplant hospital from July 1994 to July 2011. Patients were followed up until 12 July 2012. The following exclusion criteria were applied: any patient who had a previous organ transplant, had any other invasive surgery at the date of transplantation, had incomplete medical records or had died within the first 72 h. Finally, a total of 632 patients were available for study. Data were drawn from hospital medical records and transplant database. Pre-transplant variables were recorded, including age, gender, body mass index (BMI), indication for OLT, Model for End-stage Liver Disease (MELD) score, haemoglobin, haematocrit, platelet count, prothrombin time, serum total bilirubin levels, serum creatinine levels, fasting blood glucose and prior diagnosis of diabetes. In addition, the following surgical variables were studied: surgical technique; transfusion requirements; operating time; and cold ischaemia time (CIT). The postoperative period was defined as the 7 days immediately following the date of transplant. Data relating to immunosuppressive drug regimen, creatinine, insulin, parenteral nutrition (PN), continuous veno-venous haemodiafiltration (CVVHDF), use of insulin drip, and glucose were measured across the postoperative period.

This study conformed to the principles of the Helsinky Declaration, and was reviewed and approval by the Local Research Committee (code # CEIC2011/328). Written inform consent was obtained from all participants.

### Glucose measurements

Glucose was determined in fasting plasma samples by the glucose oxidase peroxidase method, using an Advia 2400 autoanalyser from Siemens Healthcare Diagnostics (Barcelona, Spain). Blood samples were taken every day at 7:00 am from a radial arterial catheter or from a peripheral venous device if the catheter had been withdrawn before day 7.

### Approach to insulin and glucose control

There was a specific protocol for the use of insulin or any specific target for glucose control during the study. Commencement of insulin was decided by the intensive care unit (ICU) medical staff, and insulin dosage was adjusted by ICU nurses with the general aim of maintaining glucose levels between 120 and 180 mg/dL (6.6 and 10 mM).

### Outcomes and definitions

Patients were classified as known diabetic patients if they had been informed of this diagnosis by a physician before admission or were on antihyperglycaemic agents, insulin or diet therapy. Patients were followed up by the study team throughout their hospital stay. After discharge, vital status data were obtained by reviewing the National Health Registry, by contacting patients or their families individually and, in cases where patients had been hospitalised, by reviewing the hospital records of major clinical events. Patient survival was defined as the period of time between transplantation and end of follow-up or death.

### Missing data management

The following variables in the data set had missing data: MELD (57%); red blood cell requirements (RBC, 0.4%); hepatitis C virus (HCV, 0.1%); height (3%); body weight (3%); creatinine levels (3%); and cytomegalovirus infection (1%). To compensate for missing data, chained equations were used to perform multiple imputations. Both complete and incomplete variables were used as predictors during the imputation process.

### Statistical analysis

Data are presented as the mean ± SD, or as median (Percentil 25, Percentil 75). Differences between groups were assessed using U Mann–Whitney test for continuous variables and Chi squared test for categorical variables.

**Fig. 1:**
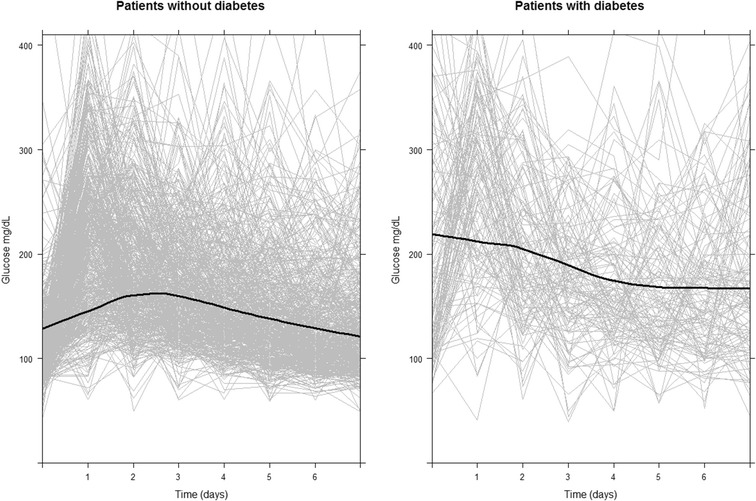
Subject-specific trends in glucose measurements and overall smooth (*loess smoothing*) trends among patients with and without diabetes

In view of the different glucose profiles obtained (see Fig. 1), independent multivariate Cox models were constructed in diabetic and nondiabetic patients. In both cases a selection of variables of known prognostic value (age, gender, BMI, etiology, MELD, creatinine, CIT, RBC and platelets transfusion, PN, and CVVHDF) were included. A backward stepwise procedure using Akaike Information Criterion was then performed to obtain a final model with the selected predictors.

The relationship between glucose profiles and the risk of death were modelled by using flexible joint modelling of longitudinal data and time-to-event analysis [13]. The idea is to construct an appropriate flexible mixed-effects model with spline smoothing for describing the evolution of glucose profiles, and then to use these estimated evolutions as a time-dependent covariate in a Cox model. The both sub-models can be represented as follows;

#### Longitudinal sub-model

$$ \begin{aligned} { \log }\left( {\text{Glucose}} \right)_{\text{i,diab}} & = \left( {\beta_{0} {\text{ + b}}_{\text{i0}} } \right) + \left( {\beta_{1} {\text{ + b}}_{\text{i1}} } \right)B_{n} \left( {{\text{t,d}}_{1} } \right) \\ &\quad + \left( {\beta_{2} {\text{ + b}}_{\text{i2}} } \right)B_{n} \left( {{\text{t,d}}_{2} } \right) + \left( {\beta_{3} {\text{ + b}}_{\text{i2}} } \right)B_{n} \left( {{\text{t,d}}_{3} } \right)  \\ &\quad + \beta_{4} {\text{age}}_{i} + \beta_{5} {\text{bmi}}_{i} + \beta_{6} {\text{carc}}_{i} \\ &\quad + \beta_{7} {\text{TH}}_{i} + \beta_{8} {\text{NPTt}}_{i} + \beta_{9} {\text{meld}}_{i} + \varepsilon_{i} \\ \end{aligned} $$

$$ \begin{aligned} { \log }\left( {\text{Glucose}} \right)_{{{\text{i,no}} - {\text{diab}}}} & = \left( {\beta_{0} {\text{ + b}}_{\text{i0}} } \right) + \left( {\beta_{1} {\text{ + b}}_{\text{i1}} } \right)B_{n} \left( {{\text{t,d}}_{1} } \right) \\ & \quad+ \left( {\beta_{2} {\text{ + b}}_{\text{i2}} } \right)B_{n} \left( {{\text{t,d}}_{2} } \right) + \left( {\beta_{3} {\text{ + b}}_{\text{i2}} } \right)B_{n} \left( {{\text{t,d}}_{3} } \right)  \\ &\quad + \beta_{4} {\text{age}}_{i} + \beta_{5} {\text{carc}}_{i} + \beta_{6} {\text{NPTt}}_{i}  \\ & \quad+ \beta_{7} {\text{bmi}}_{i} + \beta_{8} {\text{TH}}_{i} + \beta_{9} {\text{hemofiltro}}_{i} + \varepsilon_{i}  \\ \end{aligned} $$

where {*B*_*n*_(t, d_*k*_); k = 1, 2, 3} denotes a B-spline basis matrix for a natural cubic spline [14].

#### Survival sub-model

$$ h_{\text{i,diab}} \left( t \right) = {\text{h}}_{0} \left( t \right){ \exp }\left( \begin{aligned} &\lambda_{1} {\text{age}}_{i} + \lambda_{2} {\text{bmi}}_{i} + \lambda_{3} {\text{carc}}_{i} + \lambda_{4} {\text{TH}}_{i} \\ &\quad + \lambda_{5} {\text{NPTt}}_{i} + \lambda_{6} {\text{meld}}_{i} + \alpha { \log }\left( {\text{Glucose}} \right)_{i} \left( t \right) \end{aligned} \right) $$

$$ h_{{{\text{i,no}} - {\text{diab}}}} \left( t \right) = {\text{h}}_{0} \left( t \right){ \exp }\left( \begin{aligned} &\lambda_{1} {\text{age}}_{i} + \lambda_{2} {\text{carc}}_{i} + \lambda_{3} {\text{NPTt}}_{i} + \lambda_{4} {\text{bmi}}_{i} \\ &\quad + \lambda_{5} {\text{TH}}_{i} + \lambda_{6} {\text{hemofiltro}}_{i} + \alpha { \log }\left( {\text{Glucose}} \right)_{i} \left( t \right) \\ \end{aligned} \right) $$

where h_0_(t) is a Weibull baseline risk function [15].

Beside the above mentioned models, in order to compare with the joint modelling approach, we conducted a simple Cox model including a coefficient of variation (CV) of the glucose measurements during the first week post-OLT.

All the statistical analyses were carried out with the statistical software R, version 3.3.1 which is freely available at cran R webpage. For this purpose, the following packages were used: *“nlme”* for fitting linear mixed models, “*survival*” to fit Cox proportional hazards regression models, “*JM”* to perform joint models for longitudinal and time-to-event data and “*lattice*” to visualize glucose profiles. P values less than 0.05 were considered statistically significant.

## Results

### Baseline and general

The 632 patients enrolled in the study were aged 52 ± 11 years (mean ± SD), 158 (25%) were women, and 125 (20%) had a history of diabetes. Alcoholic liver disease was the most common aetiology of OLT, accounting for 386 (61%) cases. There were 130 (21%) with HCV, 33 (5%) with Hepatitis B virus, and 33 (5%) with fulminant hepatitis. Carcinoma was present in 174 (28%) patients. The median duration of follow-up was 7 years (range 1–18 years), and 218 patients (34%) died. The sample had a survival rate of 85% at 1 year and 65% at 10 years.

At 1 year of follow-up, survivors were younger than non-survivors (51 ± 11 vs. 55 ± 12 years, *P *< 0.001) and registered a lower MELD score (13 ± 6 vs. 17 ± 8, *P* < 0.001). Patients who had higher transfusion requirements and required CVVHDF registered a higher mortality, as did those with longer parenteral nutrition. Baseline and postoperative characteristics in relation to death at 1 year of follow-up are summarised in Table 1.

**Table 1:** Baseline and postoperative characteristics of patients, according to vital status (after 1-year follow-up)

Variable	Survivors at 1 year (n = 542)	Non-survivors at 1 year (n = 90)	*P* value
Age, years	51 ± 11	55 ± 12	0.001
Males, n (%)	416 (77)	58 (64)	0.012
Body mass index, kg/m^2^	26.9 ± 3.9	26.5 ± 3.4	0.288
Diabetes, n (%)	111 (20)	14 (16)	0.513
Aetiology, n (%)			
Alcohol	338 (62)	48 (53)	0.220
HCV	109 (20)	21 (23)	0.484
Malignant	143 (26)	31 (34)	0.113
Fulminant	24 (4)	9 (10)	0.021
MELD	13 ± 6	17 ± 8	< 0.001
Glucose, mg/dL	130 ± 74	128 ± 72	0.709
Creatinine, mg/dL	0.9 ± 0.5	0.9 ± 0.4	0.221
CIT, hours	7.5 ± 2.2	7.7 ± 2.2	0.330
RBC transfusion, units	5 [2, 10]	10 [4, 17]	< 0.001
PT, units	0 [0, 1]	0 [0, 2]	0.170
PN, days	4 [3, 6]	6 [4, 10]	< 0.001
CVVHDF, n (%)	60 (11)	17 (18)	0.044
Metilprednisolona	537 (99)	89 (99)	0.759
Tacrolimus	444 (83)	89 (99)	0.058
Ciclosporina	161 (30)	20 (22)	0.156

Data are expressed as mean ± SD or medians [25th percentile, 75th percentile]

Pre-operative glucose and creatinine levels are shown

*BMI* body mass index, *CIT* cold ischaemia time, *CVVHDF* continuous veno-venous haemodiafiltration, *HCV* hepatitis C virus, *MELD* Model for End-stage Liver Disease score, *PN* parenteral nutrition, *PT* platelet requirements, *RBC* red blood cell requirements

### Glucose levels, diabetes and mortality

Figure 1 shows overall glucose profiles for diabetic and non-diabetic patients. Patients with diabetes had higher levels of pre-operative glucose than did those without diabetes. Among diabetic patients, glucose levels declined gradually from day 1, whereas among non-diabetic patients glucose levels rose until reaching a peak on days 2–3 and then declined to their former levels over the course of the following days.

The main characteristics of diabetic and non-diabetic patients are shown in Table 2. Briefly, diabetic patients were older, had fewer transfusion requirements and lower MELD scores than did their non-diabetic counterparts. Alcoholic liver disease and malignant aetiology were also more frequent causes of OLT in diabetic patients.

**Table 2:** Characteristics of patients, according to diabetes status

Variable	Diabetic patients (n = 125)	Non-diabetic patients (n = 507)	*P* value
Age, years	56 ± 8	51 ± 12	< 0.001
Males, n (%)	102 (81)	372 (73)	0.074
Body mass index, kg/m^2^	27.4 ± 3.6	26.7 ± 3.8	0.029
Aetiology, n (%)			
Alcohol	88 (70)	298 (58)	0.022
HCV	28 (22)	102 (20)	0.659
Malignant	52 (41)	122 (24)	< 0.001
Fulminant	2 (2)	31 (6)	0.070
MELD	13 ± 5	14 ± 6	0.044
Glucose, mg/dL	207 ± 108	111 ± 43	< 0.001
Creatinine, mg/dL	0.9 ± 0.4	0.9 ± 0.4	0.532
CIT, hours	7.5 ± 2.6	7.4 ± 2.1	0.252
RBC transfusion, units	5 [2, 9]	6 [3, 12]	0.034
PT, units	0 [0, 1]	0 [0, 1]	0.079
PN, days	4 [3, 6]	4 [3, 6]	0.788
CVVHDF, n (%)	20 (16)	57 (11)	0.192

Data are expressed as mean ± SD or medians [25th percentile, 75th percentile]

Pre-operative glucose and creatinine levels are shown

*BMI* body mass index, *CIT* cold ischaemia time, *CVVHDF* continuous veno-venous haemodiafiltration, *HCV* hepatitis C virus, *MELD* Model for End-stage Liver Disease score, *PN* parenteral nutrition, *PT* platelet requirements, *RBC* red blood cell requirements

Patients with diabetes registered a survival rate of 89% at 1 year and 68% at 10 years, figures similar to those for patients without diabetes (86 and 65% respectively).

Among patients with diabetes, the results of the Cox proportional hazards analysis showed a significant association between mortality and age, MELD score and HCV. Among non-diabetic patients a significant increase in mortality was observed among individuals who were older or presented with malignant aetiology, more transfusion requirements and more time of parenteral nutrition.

Joint modelling showed that glucose profiles taken during the first week post-transplantation were associated with mortality among patients without diabetes (*P *= 0.022), with this association not being found among those previously diagnosed with diabetes (*P *= 0.689) (Tables 3 and 4).

**Table 3:** Survival model for patients without diabetes

	Cox model		Joint model	
	HR (95% CI)	*P* value	HR (95% CI)	*P* value
Age, years	1.02 (1.00, 1.03)	0.001	1.02 (1.00, 1.03)	0.006
BMI, kg/m^2^	0.95 (0.91, 1.00)	0.033	0.96 (0.92, 1.00)	0.080
Malignant	1.49 (1.04, 2.13)	0.034	1.59 (1.11, 2.29)	0.011
RBC, units	1.02 (1.01, 2.01)	< 0.001	1.02 (1.01, 1.03)	< 0.001
PN, days	1.01 (1.00, 1.03)	< 0.001	1.06 (1.04, 1.08)	< 0.001
CVVHDF	2.29 (1.53, 3.42)	< 0.001	1.08 (0.65, 1.78)	0.768
Glucose profiles	–	–	See Fig. 1	0.022
Glucose variability (CV)	2.71 (0.72, 10.15)	0.13	–	–

HR (95% CI) means hazard ratio (95% confidence interval)

*BMI* body mass index, *RBC* red blood cells, *PN* parenteral nutrition, *CVVHDF* continuous veno-venous haemodiafiltration, *CV* coefficient of variation

**Table 4:** Survival model for patients with diabetes

	Cox model		Joint model	
	HR (95% CI)	*P* value	HR (95% CI)	*P* value
Age, years	1.03 (0.98, 1.08)	0.048	1.07 (1.02, 1.12)	0.005
MELD score	1.02 (0.96, 1.09)	0.016	1.07 (1.01, 1.14)	0.016
HCV	2.19 (1.13, 4.23)	0.020	2.36 (1.21, 4.57)	0.010
Glucose profiles	–	–	See Fig. 1	0.689
Glucose variability (CV)	0.20 (0.01, 3.57)	0.27	–	–

The results of the joint models are expressed as hazard ratios with their corresponding confidence intervals. In any case where non-linear trends in glucose levels are a covariate in the survival process, interpretation of the coefficients of association (α) becomes compromised. Accordingly, only the levels of significance (*P* values) of the coefficients of association are shown

HR (95% CI) means hazard ratio (95% confidence interval)

*MELD* Model for End-stage Liver Disease score, *HCV* hepatitis C virus, *CV* coefficient of variation

**Fig. 2:**
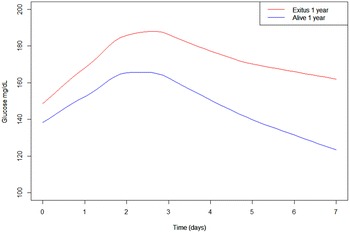
Overall smooth (*loess smoothing*) trends among patients who dead in the first year and those who did not

Regarding the interpretation of the glucose profiles, it is getting compromised. For its interpretation, we should take a look not only the intercept which is the baseline glucose level but at the profiles assessing their evolutions. As can be seen in Fig. 2, mortality is higher in individuals at higher glucose levels after 48–72 h post-OLT, also showing a worse response to the insulin treatment within the first week.

While statistically significant association between glucose and mortality is found when using glucose profiles, it is not found when using a summary measure of glucose variability such as CV.

## Discussion

In this survival study among OLT patients, the main findings were: (1) plasma glucose profiles during the first week post-OLT are different in patients with and without diabetes mellitus prior to transplantation; (2) while glucose levels are associated with a higher risk of mortality among patients without diabetes, after adjusting for potential confounding variables such as age, cause of transplantation, MELD, BMI, nutrition, and haemodiafiltration, this does not occur among patients with diabetes; and, (3) the glucose profiles analysis provides better information than a single measure of glucose variability to ascertain the relationship between glucose levels and mortality.

The fact that glucose values may give rise to an increase in long-term mortality among critical patients has been previously described. The association between glucose and mortality has been the subject of study by a number of authors [16–20], though not all report uniform results. Some authors observe that in many diseases, critical patients having higher glucose levels register higher mortality [21–24], something that has led in turn to the implementation of different protocols aimed at achieving a strict control of glycaemia, so as to maintain glucose values within the range of normality. Such strict control does not necessarily entail a decrease in mortality, however. Indeed, in some patients this has even been observed to lead to an increase in mortality, possibly due to a higher rate of hypoglycaemia [25–28].

With respect to glucose levels, there are also reports of different mortality patterns observed in patients with and without diabetes. Patients with diabetes tolerate higher glycaemic values, without repercussions on mortality, conceivably possessing some, as yet unknown, protective factors against stress hyperglycaemia. These facts, together with the finding that in the first week post-OLT diabetic patients display glucose profiles different to those of non-diabetic patients, led us to analyse these two groups of patients separately. As can be seen from Fig. 1, patients with diabetes started with higher plasma glucose values on the first day, which then gradually declined after the first 48 h. In contrast, non-diabetic patients started from appreciably lower figures which gradually rose until reaching a peak at 48–72 h, and then subsequently declined (all within the context of insulin being administered via continuous perfusion).

This agrees with the results of previous studies in which the presence of diabetes has been reported to afford a protective effect against hyperglycaemia [29–31]; hence, despite the fact that patients with diabetes display higher glycaemic values, this would not affect mortality. This might perhaps be attributable to the existence of a chronic state of hyperglycaemia in such patients, which would confer a greater tolerance to the condition and so attenuate its harmful effects in situations of stress. Already in Egi’s study, it was observed that, even within the group of patients with diabetes, those who presented with higher glycated haemoglobin figures, with worse control of their disease, and had thus chronically maintained higher glucose figures, would tolerate hyperglycaemia better [32].

Despite the many studies conducted on glycaemia in a critical care setting, very few references could be found in the field of liver transplantation [33–35], and none of them stratified patients into those with and those without diabetes. Furthermore, when glycaemic levels were studied, consideration was given only to isolated glycaemic values or summary values of variability, such as standard deviation [36] or the coefficient of variation [29], with no follow-up of glycaemia across time having been previously conducted as in our study. Moreover, joint-modelling analysis allows for a better approximation of the survival of such patients, by taking trends in glycaemic levels in the postoperative period into account.

Similarly, patients with diabetes display higher absolute glycaemic values than do patients without diabetes during the first 7 days after transplantation. Although glycaemia does not attain such high values in patients without diabetes, glycaemic levels nevertheless display a marked rise at day 3, which is absent among diabetics and could reflect glycaemic response to stress. Despite insulin being administered via continuous perfusion to maintain glycaemia figures between 120 and 180 mg/dL, the distribution adopted by glycaemia remained unchanged. This coupled with the fact that glycaemic profiles were associated with mortality solely in patients without diabetes, led us to think that the presence of this hyperglycaemic peak might possibly flag patients with the highest stress—and thus the most severe patients—and not glycaemic levels per se. This may be compatible with findings of previous studies in which correction of hyperglycaemia was observed to lead to an increase in hypoglycaemia and mortality rates. It is for this reason that, on evaluating glycaemia in post-OLT patients, we feel that account should be taken, not only of the glycaemic values but also of their trend over time, distinguishing between patients with and those without diabetes.

### Limitations of the study

The use of MELD as a severity score was introduced only from 2002 onwards thus having the measurements available in only 43% of patients. The use of this score is supported by many studies and liver transplantation societies, which consider it not only a predictor of waiting list mortality, but also a possible factor of post-OLT mortality [37], since it identifies patients with a higher risk of renal failure, perioperative bleeding and more transfusion requirements. Hence, Matthews study observed that for every 5-point rise in the MELD score there was an increase of 15 units in RBC requirements, and every 10 units of platelet concentrate transfused increased the risk of post-OLT renal failure by 5–8% and amounted to a significant increase in perioperative morbidity and mortality [38]. Although contradictory results has been found in other studies [37]. In our study, we have chosen to perform multiple imputation techniques given that traditional complete case analysis suffers from inefficiency, selection bias of subjects and other limitations. In addition, we also performed analyses estimating MELD score from prothrombin values in patients who underwent liver transplantation before 2002. Results of these analyses showed similar results from those obtained by performing multiple imputation methods (data not shown).

The time at surgery is an important predictor for the risk of death that was not included into the predictive model. In the final model, transfusion requirements; operating time, cold ischaemia time (CIT), changes in nutrition were entered. All these covariates are associated with the time of surgery which can contribute more information than the global time effect. In addition, the regression models containing all these covariates and time variable may suffer from collinearity which degrades the precision of estimate coefficients for the reason of having a strong interaction between them.

A further limitation of the study was the use of continuous insulin perfusion, which modified the glycaemic readings. Even so, it has to be said that insulin was administered to all patients in accordance with the same protocol.

This study may have clinical implications of relevance: firstly, glycaemic levels in patients with and without diabetes should not be considered in the same way, i.e., patients with diabetes appear to tolerate higher glycaemic figures without repercussions on their mortality; and secondly, trends in glucose levels during the first days post-OLT may well be of importance in terms of mortality.

## Conclusions

Glucose profiles during the first week after liver transplantation are associated with long-term mortality in patients without diabetes, after adjusting for potential confounding variables such as age, cause of transplantation, MELD, nutrition, immunosuppressive drugs, and units of insulin administered. Furthermore, these profiles are different in patients with and without diabetes.

## Abbreviations

AUC: area under the curve
BMI: body mass index
CIT: cold ischaemia time
CV: coefficient of variation
CVVHDF: continuous veno-venous haemodiafiltration
HCV: hepatitis C virus
ICU: intensive care unit
MELD: Model for End-Stage Liver Disease score
OLT: orthotopic liver transplantation
PN: parenteral nutrition
RBC: blood cell requirements
ROC: receiving operating curves
SD: standard deviation
